# Early‐Onset Thiazide‐Induced Hyponatremia Leading to Seizure in a Middle‐Aged Woman Using Aldactazide

**DOI:** 10.1155/crin/9991770

**Published:** 2026-02-25

**Authors:** Mehmet Zafer Aydın, Mehmet Kamil Teber, Zülfiye Kuzu, Mustafa Gök

**Affiliations:** ^1^ Department of Cardiology, Kayseri City Training and Research Hospital, Kayseri, Türkiye

**Keywords:** acute symptomatic hyponatremia, Aldactazide, drug-induced electrolyte disorder, hydrochlorothiazide, hyponatremia, middle-aged woman, seizure, thiazide diuretics

## Abstract

**Background:**

Thiazide‐induced hyponatremia (TIH) is a well‐recognized adverse effect of thiazide diuretics, typically occurring in elderly individuals. Severe acute hyponatremia presenting with seizures in middle‐aged adults is uncommon. Combination therapy with spironolactone and hydrochlorothiazide (Aldactazide) may increase susceptibility to rapid electrolyte shifts. We report a case of early‐onset, symptomatic hyponatremia leading to generalized seizure shortly after initiation of Aldactazide.

**Case Presentation:**

A 52‐year‐old woman with hypertension presented after experiencing a generalized tonic–clonic seizure at home. She had started Aldactazide (spironolactone + hydrochlorothiazide 25 mg daily) 3 days earlier, in addition to perindopril/amlodipine and captopril. Two weeks before symptom onset, her serum sodium level was 131 mmol/L. On admission to the intensive care unit, she was alert and hemodynamically stable, with a Glasgow Coma Scale score of 15. Laboratory evaluation revealed severe hyponatremia (115 mmol/L), hypokalemia (3.3 mmol/L; ionized 2.7 mmol/L), and hypochloremia (76 mmol/L). Lactate was transiently elevated at 8.3 mmol/L, consistent with postictal physiology. Renal, thyroid, and adrenal functions were normal. Brain CT and MRI showed no acute abnormalities. Controlled correction with 3% hypertonic saline resulted in progressive normalization of serum sodium (115 ⟶ 121 ⟶ 125 ⟶ 131 mmol/L) without overcorrection. The patient experienced complete neurological recovery and was transferred to the nephrology ward for continued monitoring. Aldactazide was discontinued.

**Conclusion:**

This case demonstrates that TIH can develop rapidly in middle‐aged individuals and may lead to life‐threatening neurological complications, including seizures. Clinicians should closely monitor serum sodium during the early phase of thiazide‐containing diuretic therapy, particularly when combination regimens such as Aldactazide are prescribed.

**Plain Language Summary**
• This case report describes a 52‐year‐old woman who developed a very low sodium level only three days after starting a medication called Aldactazide, which contains two diuretics commonly used to treat high blood pressure. Her sodium dropped quickly from 131 to 115 mmol/L, which caused a generalized seizure at home. When she arrived at the hospital, doctors confirmed severe hyponatremia (dangerously low sodium) and treated her with a special salt solution (3% hypertonic saline). Her sodium level slowly returned to normal, and she recovered fully without further seizures.
• Low sodium caused by thiazide diuretics usually happens in older patients, but this case shows that it can also occur in middle‐aged adults. It can develop very quickly and may lead to life‐threatening symptoms. The case highlights the importance of checking sodium levels soon after starting diuretic medicines like Aldactazide.

## 1. Introduction

Thiazide diuretics are among the most common causes of drug‐induced hyponatremia and are widely prescribed for the management of hypertension and edema [1, 2]. Thiazide‐induced hyponatremia (TIH) classically occurs in elderly, low‐body‐weight women and may present with nonspecific symptoms such as fatigue, nausea, or confusion [1, 3]. Severe acute hyponatremia with neurological complications—including seizures—is uncommon and typically associated with a rapid and substantial decline in serum sodium concentration [1, 4]. Reports of TIH presenting with seizures in middle‐aged adults remain rare [2, 4].

Aldactazide, a fixed‐dose combination of spironolactone and hydrochlorothiazide, is frequently used for hypertension management; however, its dual natriuretic effect may increase susceptibility to rapid electrolyte shifts in predisposed individuals [1, 2]. We present a case of early‐onset, acute symptomatic hyponatremia leading to a generalized tonic–clonic seizure in a 52‐year‐old woman after only three days of Aldactazide therapy.

## 2. Case Presentation

A 52‐year‐old woman with a history of hypertension presented to the emergency department after experiencing a generalized tonic–clonic seizure at home. Postictal confusion and transient memory loss were noted. Three days prior, she had started Aldactazide (spironolactone + hydrochlorothiazide 25 mg once daily) in addition to perindopril/amlodipine and captopril. Her serum sodium was 131 mmol/L 2 weeks before symptom onset.

On ICU admission, she was alert and hemodynamically stable (BP 121/71 mmHg, HR 53 bpm, RR 12/min, SpO_2_ 97% on room air). Neurological examination showed a GCS of 15 with no focal deficits. Systemic examination was unremarkable.

Laboratory evaluation revealed severe hyponatremia (Na^+^ 115 mmol/L), hypokalemia (3.3 mmol/L; ionized K^+^ 2.7 mmol/L), and hypochloremia (76 mmol/L). Lactate was transiently elevated (8.3 mmol/L), consistent with postictal physiology (Table 1). Renal function and serum osmolality were preserved; thyroid function and morning cortisol levels were normal. Urinalysis revealed low specific gravity without proteinuria. CT and MRI of the brain showed no acute pathology.

**TABLE 1:** Laboratory values before admission, at presentation, and after treatment.

Parameter	Reference range	Before admission	At admission	After treatment
Sodium (mmol/L)	135–145	131	115–121	135–138
Potassium (mmol/L)	3.5–5.1	4.2	3.3 (ionized 2.7)	3.7–4.1
Chloride (mmol/L)	98–107	100	76	96–98
Creatinine (mg/dL)	0.6–1.2	0.73	0.91	0.75–0.82
eGFR (mL/min/1.73 m^2^)	> 60	95	73	83–103
Lactate (mmol/L)	0.5–2.2	—	8.3	1.6
TSH (mIU/L)	0.27–4.2	—	0.72	—
Cortisol (μg/dL)	6.0–18.4	—	15.2	—
CRP (mg/L)	0–5	1.1	0.3	1.2–2.6
Urine specific gravity	1.005–1.030	—	1.006	—

*Note:* This table summarizes baseline, admission, and posttreatment laboratory findings, highlighting the progression from mild chronic hyponatremia to severe acute electrolyte disturbance and subsequent normalization following appropriate management. Reference ranges correspond to institutional laboratory standards.

Controlled correction with 3% hypertonic saline was initiated. Sodium improved gradually from 115 ⟶ 121 ⟶ 125 ⟶ 131 mmol/L without overcorrection. She remained neurologically stable with no recurrence of seizures. Hemodynamic parameters remained normal throughout the ICU stay. She was transferred to the nephrology service for further monitoring and discharged with complete neurological recovery. Aldactazide was discontinued permanently.

## 3. Discussion

TIH is a well‐recognized adverse effect of thiazide diuretics and is classically described in elderly, low–body weight women [1, 3]. However, this case illustrates that severe, life‐threatening hyponatremia may develop rapidly—even within a few days—after thiazide initiation in middle‐aged individuals, particularly when multiple predisposing factors coexist [1, 2]. Importantly, this report underscores that the presence of preexisting hyponatremia represents a critical vulnerability that can precipitate a disproportionate and rapid decline in serum sodium after diuretic exposure [1, 2].

The pathophysiology of TIH involves inhibition of sodium reabsorption in the distal convoluted tubule, leading to impaired free‐water excretion and a functional state resembling the syndrome of inappropriate antidiuretic hormone secretion (SIADH) [1, 3]. This mechanism is exacerbated by low solute intake, which limits the kidney’s capacity to excrete free water and promotes dilutional hyponatremia [1, 5]. In our patient, urinalysis demonstrated a markedly low urine specific gravity (1.004), indicating dilute urine. Although thiazides typically impair urinary dilution, early‐onset TIH may still present with low specific gravity in the setting of reduced osmotic load and increased free‐water intake [1, 3, 5]. Urine sodium and osmolality were not available at presentation due to the urgent need for seizure management; this limitation is acknowledged.

A defining feature of this case is that the patient was already mildly hyponatremic (serum sodium 131 mmol/L) prior to initiation of Aldactazide. Mild chronic hyponatremia is often clinically silent but reflects impaired renal water handling and reduced physiological reserve [2, 4]. In such patients, the introduction of thiazide‐containing diuretics may trigger abrupt progression to severe, symptomatic hyponatremia with serious neurological consequences, including seizures [1, 4, 6]. This observation represents the central teaching point of the case.

Dietary factors further modulate susceptibility to TIH. Low sodium intake, frequently recommended for hypertension management, reduces effective solute excretion and increases the risk of hyponatremia in patients receiving thiazide diuretics [1, 5]. The patient reported adherence to a low‐salt diet, which likely contributed to the rapid decline in serum sodium. In contrast, adequate potassium balance may mitigate TIH severity by limiting intracellular sodium shifts [1, 7]. These considerations highlight the importance of individualized dietary counseling when initiating thiazide therapy, particularly in patients with baseline hyponatremia or those receiving other medications known to promote hyponatremia [3].

Electrolyte disturbances beyond sodium also play a role. The patient presented with hypokalemia, a recognized independent risk factor for TIH [1, 7]. Potassium depletion promotes intracellular potassium loss in exchange for sodium entry into cells, thereby exacerbating extracellular hyponatremia and increasing the risk of neurological manifestations [7]. The coexistence of hypokalemia supports a diuretic‐mediated mechanism and emphasizes the need for early electrolyte monitoring.

Drug dosage is another modifiable risk factor. The patient received hydrochlorothiazide 25 mg daily as part of a fixed‐dose combination. Evidence suggests that lower starting doses (e.g., 12.5 mg) provide comparable antihypertensive efficacy while substantially reducing the risk of hyponatremia [1, 8]. Higher doses may disproportionately increase adverse electrolyte effects without additional clinical benefit. Conservative dosing is therefore especially important in patients with preexisting hyponatremia.

With respect to timing, most cases of TIH occur within the first 30 days of therapy, and severe symptomatic presentations such as seizures or coma are predominantly reported in elderly patients treated for several days to weeks [1, 3]. Nevertheless, rare cases of very early onset hyponatremia within 2–5 days have been described, particularly in susceptible individuals with baseline hyponatremia or low solute intake [1, 4, 6]. Our case represents an uncommon but clinically important example of severe symptomatic hyponatremia with seizure occurring within 3 days in a nonelderly adult.

Causality assessment using the Naranjo Adverse Drug Reaction Probability Scale yielded a score of 7, indicating a probable causal relationship between Aldactazide exposure and the observed severe hyponatremia [9]. The close temporal association, absence of alternative etiologies, objective biochemical confirmation, and prompt improvement after drug discontinuation support this conclusion.

Prompt recognition and controlled correction of serum sodium are essential to prevent permanent neurological injury. In this patient, cautious administration of hypertonic saline resulted in gradual normalization of serum sodium without overcorrection or osmotic demyelination, consistent with current guideline recommendations for the management of acute symptomatic hyponatremia [4].

## 4. Conclusion

Acute symptomatic hyponatremia can develop rapidly after starting thiazide‐containing medications such as Aldactazide, even in middle‐aged individuals without classical risk factors. Severe sodium decline may lead to life‐threatening complications, including generalized seizures. Clinicians should monitor serum sodium closely during the first days of therapy and maintain a high index of suspicion for TIH in patients presenting with acute neurological symptoms shortly after starting a diuretic.

## Funding

No funding was received for this manuscript.

## Consent

Written informed consent was obtained from the patient for publication of this case report and any accompanying clinical information.

## Conflicts of Interest

The authors declare no conflicts of interest.

## Data Availability

The data that support the findings of this study are available on request from the corresponding author. The data are not publicly available due to privacy or ethical restrictions.
